# Effects of Microbubble Size on Ultrasound-Induced Transdermal Delivery of High-Molecular-Weight Drugs

**DOI:** 10.1371/journal.pone.0138500

**Published:** 2015-09-21

**Authors:** Ai-Ho Liao, Hsin-Chiao Ho, Yi-Chun Lin, Hang-Kang Chen, Chih-Hung Wang

**Affiliations:** 1 Graduate Institute of Biomedical Engineering, National Taiwan University of Science and Technology, Taipei, 10607, Taiwan; 2 Department of Medical Engineering, National Defense Medical Center, Taipei, 11490, Taiwan; 3 Department of Otolaryngology-Head and Neck Surgery, Tri-Service General Hospital, National Defense Medical Center, Taipei, 11490, Taiwan; 4 Graduate Institute of Medical Sciences, National Defense Medical Center, Taipei, 11490, Taiwan; 5 Hualien Armed Forces General Hospital, Hualien County, 97144, Taiwan; University of California, Merced, UNITED STATES

## Abstract

The transdermal delivery of a wide range of high-molecular-weight drugs is limited by the stratum corneum layer of the epidermis representing a significant barrier to penetration across the skin. This study first determined the different effects of different-size ultrasound (US) contrast agents and microbubbles (MBs) for enhancing the transdermal delivery of high-molecular-weight drugs. The effects of US-mediated different-size (1.4, 2.1, and 3.5 μm) MBs (as a contrast agent) and ascorbyl tetraisopalmitate (VC-IP) on enhancing skin transdermal delivery were demonstrated both *in vitro* and *in vivo*. The results indicated that at a power density of 3 W/cm^2^ the penetration depth in group US combined with 3.5-μm MBs and penetrating VC-IP (U+3.5) was 34% and 14% higher than those in groups US combined with 1.4-μm MBs and penetrating VC-IP (U+1.4) and US combined with 2.1-μm MBs and penetrating VC-IP (U+2.1), respectively, for the agarose phantoms, while the corresponding increases for pigskin were 37% and 19%.In terms of the skin permeation of VC-IP, the VC-IP concentration in group U+3.5 was 23% and 10% higher in than those in groups U+1.4 and U+2.1, respectively. The whitening effect (luminosity index) of mice skin in group U+3.5 had increased (significantly) by 28% after 1 week, by 34% after 2 weeks, and tended to stabilize after 3 weeks (45%) in C57BL/6J mice over a 4-week experimental period. The results obtained in this study indicate that combining US with MBs of different sizes can produce different degrees of skin permeability so as to enhance the delivery of VC-IP to inhibit melanogenesis, without damaging the skin in mice.

## Introduction

Drugs can be administered to the body via the oral route, transdermally, or by inhalation or intravenous injection. Transdermal drug delivery (TDD) offers several advantages over the conventional methods of drug administration such as avoiding first-pass metabolism, reducing systemic side effects, and providing sustained release of drugs [1]. However, ensuring that drugs permeate across the skin barrier remains a major challenge [2]. Various approaches have been developed in recent decades to overcome the barrier represented by the skin, including physical, chemical and biochemical approaches using iontophoresis, sonophoresis, microneedles, penetration enhancers, liposomal vesicles, and enzyme inhibition [3]. The bioavailability limitations associated with topical and transdermal application routes mean that the currently available transdermal medicines are limited to small molecules (<500 Da) [4]. Sonophoresis has recently been used with commercially available lipid microbubbles (MBs) to enhance TDD [4]. However, TDD of large molecules (>500 Da) remains limited by the upper layer of the skin [the stratum corneum (SC)] impeding the flux of exogenous molecules across it and thereby representing a strong barrier to the permeation of most drugs into deeper dermal layers [3]. Recently, sonophoretic TDD has been used with lipid MBs to enhance the delivery of the optical clearing agent into porcine skin via cavitation [5]. The feasibility of controlled cavitation using commercial lipid MBs at high frequency was explored in a rat model [6]. However, the optimize MB size and efficiency of this method for TDD of larger moleculars are still unclear.

Similar to TDD for the skin, the blood–brain barrier (BBB) is well known as being the main obstacle for successfully delivering drugs into the brain parenchyma. The combination of focused ultrasound (US) and MBs has been shown to be the most promising approach to achieving localized BBB opening without damaging the surrounding tissue [7–9]. This method of BBB opening was found to increase the permeability by at least a 100-fold [7]. Other studies have demonstrated that BBB opening can be induced safely using nonlinear MB oscillation at a certain pressure threshold, with the volume of drug administered being highly dependent on both the power density and MB diameter [10, 11]. Moreover, our previous study found that larger MBs were more resistant to US destruction and that they enhanced the transfection efficiency of auditory hair cells at a constant US power density [12].

Vitamin C is a natural antioxidant whose topical application ameliorates the effects of oxidative stress such as DNA damage caused by exposure of the skin to ultraviolet (UV) radiation. However, the efficacy of vitamin C when it is applied directly to the skin is limited by both its poor penetration of the skin and its instability in formulations. A lipophilic vitamin C derivative, ascorbyl tetraisopalmitate (VC-IP), was recently produced and applied to UV-induced skin pigmentation with the aim of determining its potential as a more valid form of vitamin C [13]. That study found that VC-IP can enhance cellular tolerance against ultraviolet-B (UVB) radiation and reduce the production of interleukin-1alpha (IL-1α) and prostaglandin E2 (PGE_2_) in UVB-irradiated keratinocytes and suppress melanocyte proliferation [13]. However, the average molecular weight of VC-IP is 1129.8 Da, which makes it too large for TDD. Low-frequency sonophoresis can allow nanoparticles as large as around 100 nm to penetrate the SC and enter the viable epidermal and dermal skin layers [14]. However, US alone cannot provide sufficient permeability enhancement for the delivery of large molecules [15]. There are many reports on the delivery of large molecules using microneedle patches, but this approach constitutes a minimally invasive method.

With the aim of improving the efficiency VC-IP TDD, the present study evaluated the impact of combining US with MBs of different sizes on the efficiency of TDD for large molecules both *in vitro* and *in vivo*.

## Materials and Methods

### Preparation of albumin-shelled MBs of different sizes

Albumin MBs were prepared according to the procedure used in our previous study [12, 16]. Dextrose [D(+)-Glucose, Acros Organics, Fair Lawn, NJ, USA] was purchased to prepare stock solutions of 5%, 20%, and 45% (w/v) dextrose in phosphate buffered saline (PBS; pH 7.4, 0.9% sodium chloride). Human serum albumin (HSA) was purchased as a sterile 20% solution (Octapharma, Vienna, Austria), which was diluted with PBS to make stock solutions containing 1.32% (w/v) HSA. Briefly, 1.4-μm, 2.1-μm, and 3.5-μm MBs were generated by sonication in 10 ml of solution mixing the 1.32% albumin with 5%, 20%, and 45% dextrose, respectively, in perfluorocarbon gas in PBS using a sonicator (200 W; Branson Ultrasonics, Danbury, CT, USA) for 2 min. The number of perfluorocarbon-filled albumin MBs in the solution was measured with an electrical-sensing-zone device (MultiSizer III, Beckman Coulter, Fullerton, CA, USA) using a 30-μm-aperture probe with measurement boundaries of 0.6–20 μm. The size distribution in the suspension was measured based on dynamic light scattering (Zetasizer Nano ZS90, Malvern Instruments, Worcestershire, UK).

### Measurements of penetration depth in agarose phantoms

**Fig 1:**
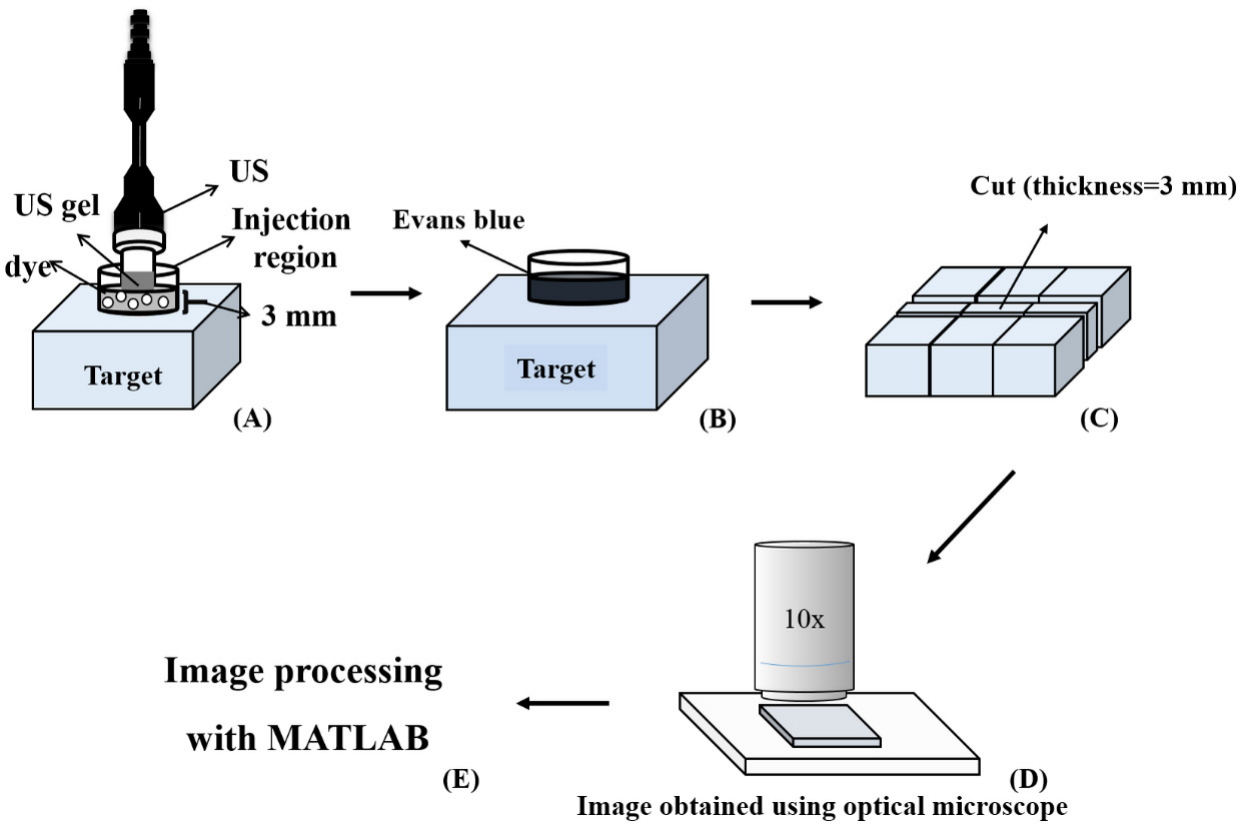
Schematic diagram of the system setup for measuring the penetration depth in agarose phantoms.

Fig 1 shows a schematic diagram of the system setup. The model drug, Evans blue (0.1 mg; 960.81 Da; E2129, Sigma-Aldrich, St Louis, MO, USA), was dissolved in 10 ml of PBS and then stirred for 1 hour at 4°C. Circular 0.3%-agarose phantoms were constructed with a radius of 1.2 cm and a height of 3 mm (encircled with US gel to prevent leakage); the round area of each phantom was loaded with Evans blue or MBs. The probe of the sonoporation gene transfection system (ST 2000V, NepaGene, Ichikawa, Japan) was positioned 3 mm from the top of the phantom. After adding 500 μl of the 1.4-μm, 2.1-μm, and 3.5-μm MBs, the area was sonicated by the 1-MHz US transducer of the sonoporation system successively at the following acoustic power densities: 1 W/cm^2^ for 1 min, 2 W/cm^2^ for 1 min, and 3 W/cm^2^ for 1 min. The duty cycle was set at 50% and a 1.2-cm-diameter transducer was used. The change in temperature during US sonication at power densities of 2 W/cm^2^ and 3 W/cm^2^ for 1 min in 37°C did not exceed 0.3°C, as measured by a thermometer (Optris LS, Optris, Berlin, Germany). The MBs were subsequently removed from the surface and the area was washed three times for 1 min each with PBS. The Evans blue solution was then injected into the area on the phantom and left there for 5 min. The Evans blue was removed and the area was again washed three times for 1 min each with PBS. Sections (3 mm thick) of the phantom were cut and prepared for light-microscopy evaluation. The penetration depths of the Evans blue were measured using MATLAB (The MathWorks, Natick, MA, USA). The light-microscopy images were converted into 8-bit grayscale images and image histogram-based binarization was performed [17]. For histogram-based binarization, the peak-and-valley thresholding of the target was performed based on the histogram representing the data from many experiments [17]. The boundary was then detected using Sobel-operator-based edge detection [18], with the same threshold used when processing all of the images. Finally, the area of the penetration region was measured, and this was divided by the length of the *x*-axis of the image to obtain the mean penetration depth (*y*-axis) of the Evans blue.

### Measurements of penetration depth in pigskin

Fresh porcine skin was obtained from the Affiliated Slaughterhouse of New Taipei City Meat Market. The protocol for the experiments involving pigskin was similar to that described for the agarose phantom. The treatment area on a 2-mm-thick sample of pigskin was sonicated by the 1-MHz US transducer of the sonoporation system successively at power densities of 1 W/cm^2^ for 1 min, 2 W/cm^2^ for 1 min, and 3 W/cm^2^ for 1 min after adding 500 μl of the three different sizes of MBs. The MBs were removed and the area was washed three times for 1 min each with PBS. The Evans blue solution was then applied to the area on the pigskin and left there for 15 min. The Evans blue was then removed from the surface and the area was washed three times for 1 min each with PBS. The treated areas of pigskin were removed and then embedded with an optimal-cutting-temperature solution (Surgipath FSC 22, Leica Microsystems, Buffalo Grove, IL, USA) on round specimen disks with a diameter of 2.2 cm. The embedded samples were placed on the –25°C freezing stage of a cryostat (Microm HM550 series, Thermo, Braunschweig, Germany) for about 30 min. Transverse sectioning was performed at a slice thickness of 7 μm. Sections attached to the microscopy slides were air-dried at room temperature and mounted for microscopy examination. The average penetration depth was measured by sampling 10 depths in each slide.

### *In vitro* skin penetration by VC-IP

C57BL/6J mice weighing 20–25 g were killed by CO_2_ asphyxiation. The dorsal hairs were shaved with clippers and a full-thickness skin specimen was surgically removed, carefully cleaned with PBS, and cut into square pieces (2 cm × 2 cm). The *in vitro* skin penetration was tested using static Franz diffusion cells over an area of 2.14 cm^2^ according to the experimental design used in our previous study [19]. The temperature of the diffusion assembly was maintained at 37°C. The probe of the sonoporation system (3 W/cm^2^ for 1 min) and MBs in PBS or 3% (v/v) VC-IP (average molecular weight of 1129.8 Da; Wako Pure Chemicals, Tokyo, Japan) in olive oil (400 μl) (as a control) were applied to the donor cells facing the SC side of the skin, and occluded with Parafilm (Pechiney Laboratory Safety Products and Apparel, Chicago, IL, USA). The receptor diffusion half cell facing the dermis side was filled with PBS (pH 7.4, 12 ml); the cell contained a stirring bar rotating at 600 rpm and 0.01% gentamicin to prevent bacterial degradation of the VC-IP during the penetration process. Instead of MBs, the solutions in the diffusion cell were filtered through a 0.2-μm micropore filter (Nalgene, Rochester, NY, USA) or a 0.22-μm micropore filter (Millex, Darmstadt, Germany). Aliquots (200 μl) of receptor solution were taken after various time intervals (0, 4, 8, 12, 16, 24, 26, 29, and 32 hours), with the cell refilled each time with the same volume of fresh receptor solution. Samples were kept in a freezer until analyzed by a UV/visual spectrophotometer (Lambda 40, Perkin Elmer Ltd, Bridgeville, PA, USA).

At the end of the penetration experiments (i.e., after 32 hours), the skin sample was detached from the diffusion cells and carefully rinsed five times with distilled water to remove excess VC-IP from its surface. The skin was cut into 0.1-g pieces and homogenized with 1 ml of receptor solution for 2 min at 10,000 rpm (Polytron-Aggregate PT3100, Kinematica, Luzern, Switzerland). The homogenized suspension was centrifuged for 20 min at 3,100×*g* (Thermo Fisher Scientific, Bremen, Germany) and then the concentrations of VC-IP in the supernatant were determined by the UV/visual spectrophotometer. Sample volumes of 200 μl were added to the cuvette and place in the spectrophotometer. The VC-IP calibration curve served as the standard curve against which the absorption peaks and the corresponding concentrations of VC-IP in the samples were measured.

### Animal treatments

The melanin content of origanoside was investigated in the C57BL/6J mouse model [20]. Five-week-old mice weighing 20–25 g were obtained from Bio Lasco (Taipei, Taiwan). The experimental protocol was approved by the Institutional Animal Care and Use Committee of the National Defense Medical Center, Taipei, Taiwan. Animals were cared for in compliance with institutional guidelines and regulations. Throughout the experiments the animals were housed in stainless-steel cages in an air-conditioned room with the temperature maintained at 25–28°C and with alternating light and dark periods of 12 hours each. The animals were acclimatized for 7 days prior to the experiment. The hair was then removed and the skin color was measured using a Chroma Meter (CR-400, Konica Minolta Sensing, Tokyo, Japan) before exposure to UVB irradiation (G8T5E, Sankyo, Tokyo, Japan) to induce hyperpigmentation (total energy dose per exposure = 1 J/cm^2^, wavelength = 306 nm, three times weekly for 2 weeks), after which the skin color was measured again. The animals were divided into the following six groups (*n* = 5 per group, treatment applied three times weekly for 4 weeks, the interruption time interval ranged between 1 and 2 days (mean 1.3 day)): (1) no treatment (group C), (2) penetrating VC-IP alone (group D), (3) US combined with penetrating VC-IP (group U), (4) US combined with 1.4-μm MBs and penetrating VC-IP (group U+1.4), (5) US combined with 2.1-μm MBs and penetrating VC-IP (group U+2.1), and (6) US combined with 3.5-μm MBs and penetrating VC-IP (group U+3.5). The US was applied at 1 W/cm^2^, 2 W/cm^2^, or 3 W/cm^2^ for 1 min (except when stated otherwise, the power density was 3 W/cm^2^), and 3% (0.5 ml/cm^2^) VC-IP was used in all cases. The change in skin color induced by each of the treatments was assessed at predetermined times using the Chroma Meter. The luminosity index, *L* [21], was calculated on each measurement day before and after treatment.

### Histochemistry

Skin tissue samples (approximately 8 mm × 8 mm) were cut from the treatment area immediately after the experiments and stored in a 10% formalin solution. Hematoxylin and eosin (Sigma-Aldrich) staining was applied, and the samples were analyzed by an expert histologist.

### Statistical analysis

The obtained data were analyzed statistically using *t*-test and multiple-comparison Bonferroni procedure. A probability value of *p*<0.05 was considered indicative of a significant difference. Data are presented as mean±SD values.

## Results

### Evaluations of the penetration depth in agarose phantoms

**Fig 2:**
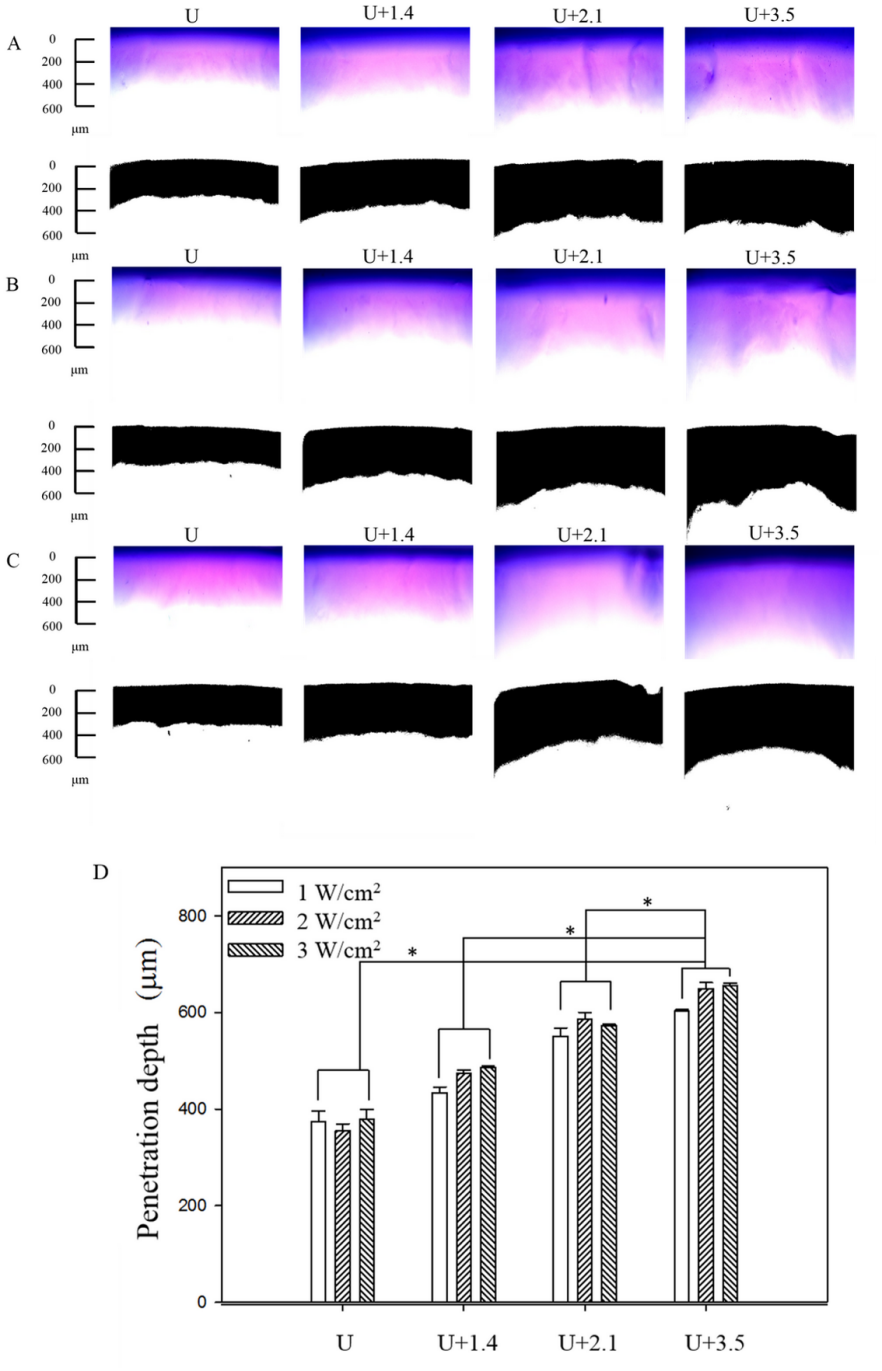
Microscopy images of the agarose phantoms obtained before (upper row) and after (lower row) MATLAB-based image processing for combining US at 1 W/cm^2^ (A), 2 W/cm^2^ (B), and 3 W/cm^2^ (C) with 1.4-μm, 2.1-μm, and 3.5-μm MBs after the Evans blue solution was allowed to stand for 1 min. (D) Quantification of the penetration depths in the three groups. Asterisk, *p <* 0.05; data are mean and SD values.

Fig 2A–2C show microscopy images of the agarose phantoms obtained before (upper row) and after (lower row) MATLAB-based image processing for combining US at 1 W/cm^2^, 2 W/cm^2^, and 3 W/cm^2^ with 1.4 μm, 2.1 μm, and 3.5 μm MBs (*n* = 4) after the Evans blue solution was allowed to stand for 1 min. Fig 2D quantifies the penetration depths in the various groups. The penetration depth was greater for larger MBs when applying US at the same power density, but it did not differ significantly when using MBs of the same size and different US power densities. While the penetration depth did not differ significantly between 2 W/cm^2^ and 3 W/cm^2^ US (*p*>0.05), the penetration was more uniform in the latter condition for all sizes of MBs tested. The penetration depths were 28%, 51%, and 73% greater in groups U+1.4, U+2.1, and U+3.5 with 3 W/cm^2^ US than in group U, respectively (Bonferroni *p<*0.05): the penetration depth was 655±5 μm in group U+3.5, compared to 379±20 μm, 486±3 μm, and 573±2 μm in groups U, US+1.4, and US+2.1, respectively. These results in Evans blue ate that the uniformity and the enhancement of the penetration depth were both greatest for 3 W/cm^2^ US combined with 3.5 μm MBs.

### Evaluations of the penetration depth in pigskin

**Fig 3:**
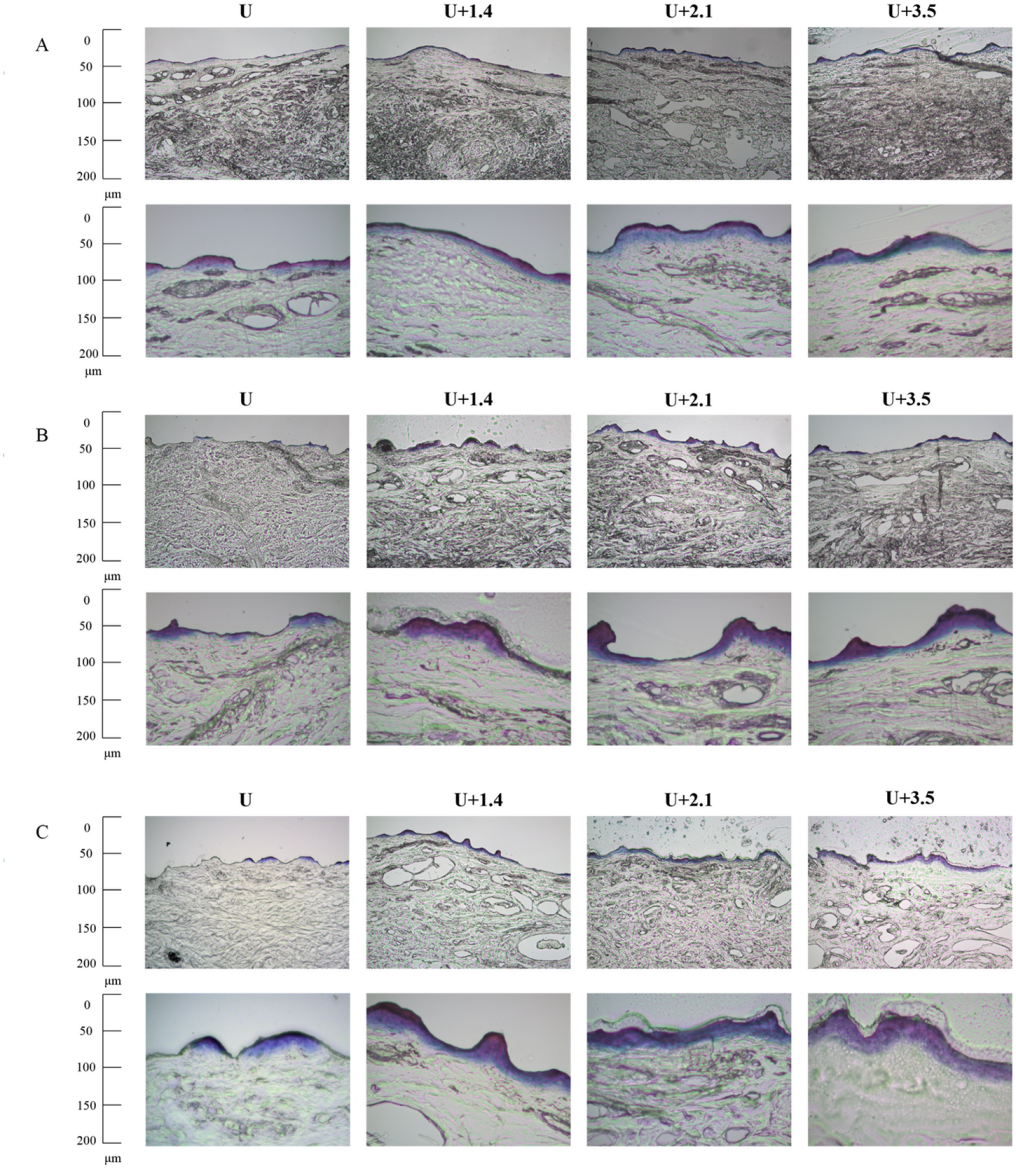
Representative histological sections of pigskin in groups U, U+1.4, U+2.1, and U+3.5 after US treatment at 1 W/cm^2^ (A), 2 W/cm^2^ (B), and 3 W/cm^2^ (C).

**Fig 4:**
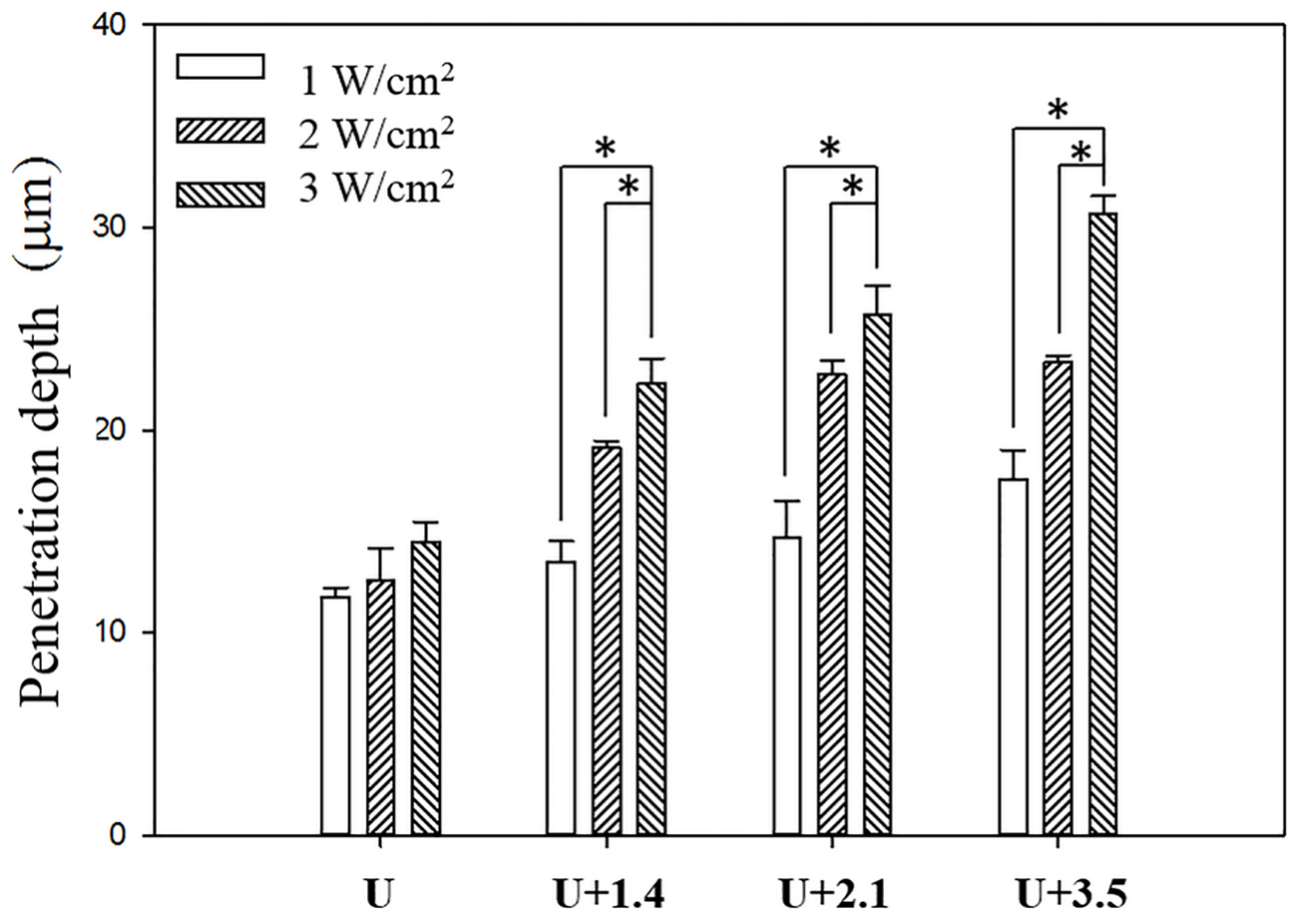
Quantification of the penetration depths for pigskin in the various groups. Asterisk, *p <* 0.05; data are mean and SD values.

The pigskin samples in groups U, U+1.4, U+2.1, and U+3.5 after treatment with US at 1 W/cm^2^ (Fig 3A), 2 W/cm^2^ (Fig 3B), and 3 W/cm^2^ (Fig 3C) were then cryosectioned for light-microscopy evaluation at magnifications of ×100 (upper row) and ×400 (lower row) (Primo Star, Zeiss-Jena, Jena, Germany). Fig 4 quantifies the penetration depths in the four groups (*n* = 4). The degree of penetration in both the cuticle and the epidermis was significantly greater for 2 W/cm^2^ and 3 W/cm^2^ US than for 1 W/cm^2^ US (Bonferroni *p<*0.05), and was greatest in group US+3.5 with 3 W/cm^2^ US. The overall penetration depth in group U with 3 W/cm^2^ US was 14.5±1.0 μm, and this increased to 22.3±1.24 μm, 25.7±1.43 μm, and 30.6±0.9 μm in groups US+1.4, US+2.1, and US+3.5, but with no significant difference between groups US+1.4 and US+2.1 (*p*>0.05). The uniformity was also greatest for group US+3.5 with 3 W/cm^2^ US, and so this condition was used for the subsequent experiments on *in vitro* skin penetration and *in vivo* animal treatments.

### *In vitro* skin penetration by VC-IP

**Fig 5:**
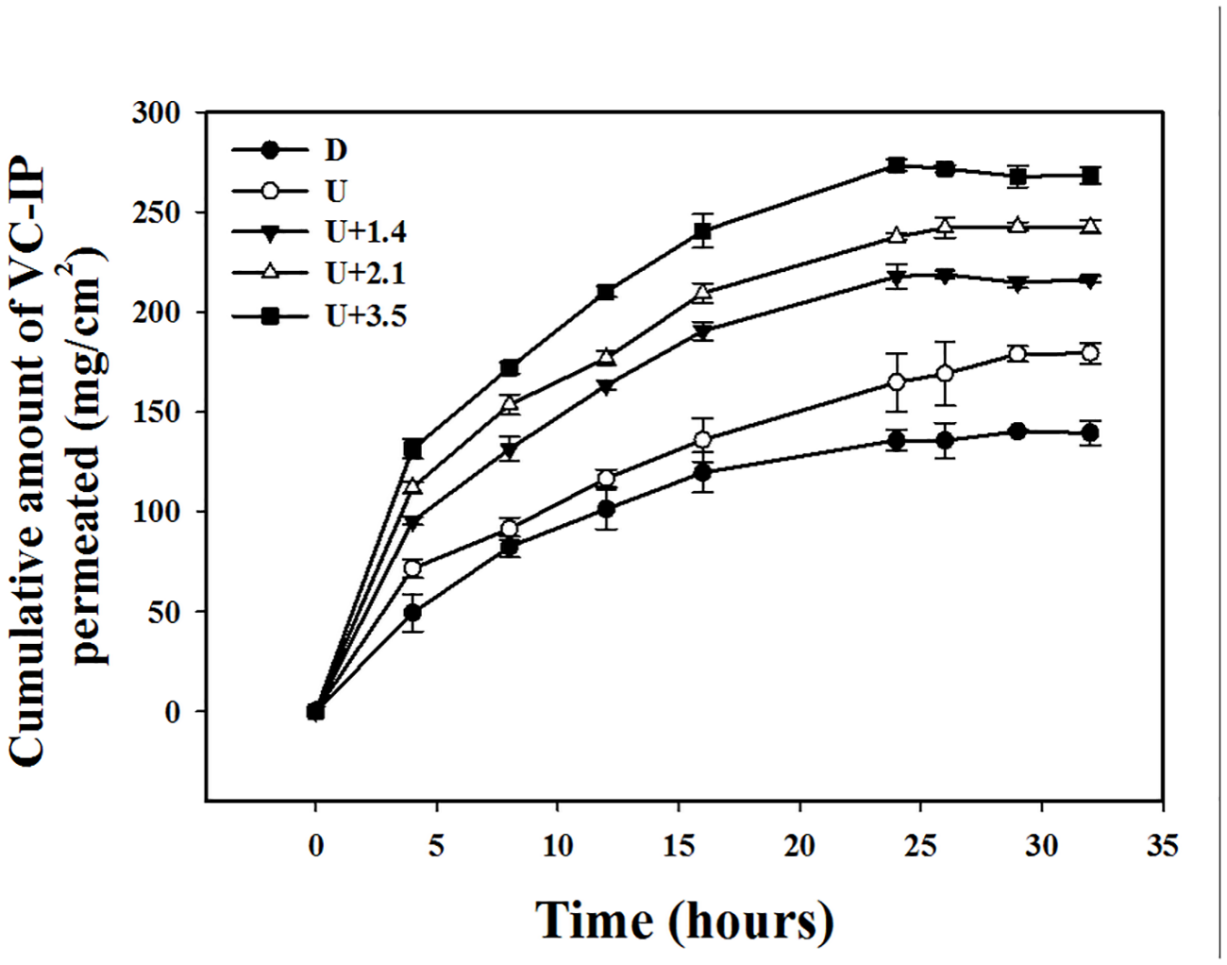
VC-IP concentrations in groups C, U+1.4, U+2.1, and U+3.5 for percutaneous penetration over 32 hours as analyzed using the UV/visual spectrophotometer. Data are mean and SD values.

**Fig 6:**
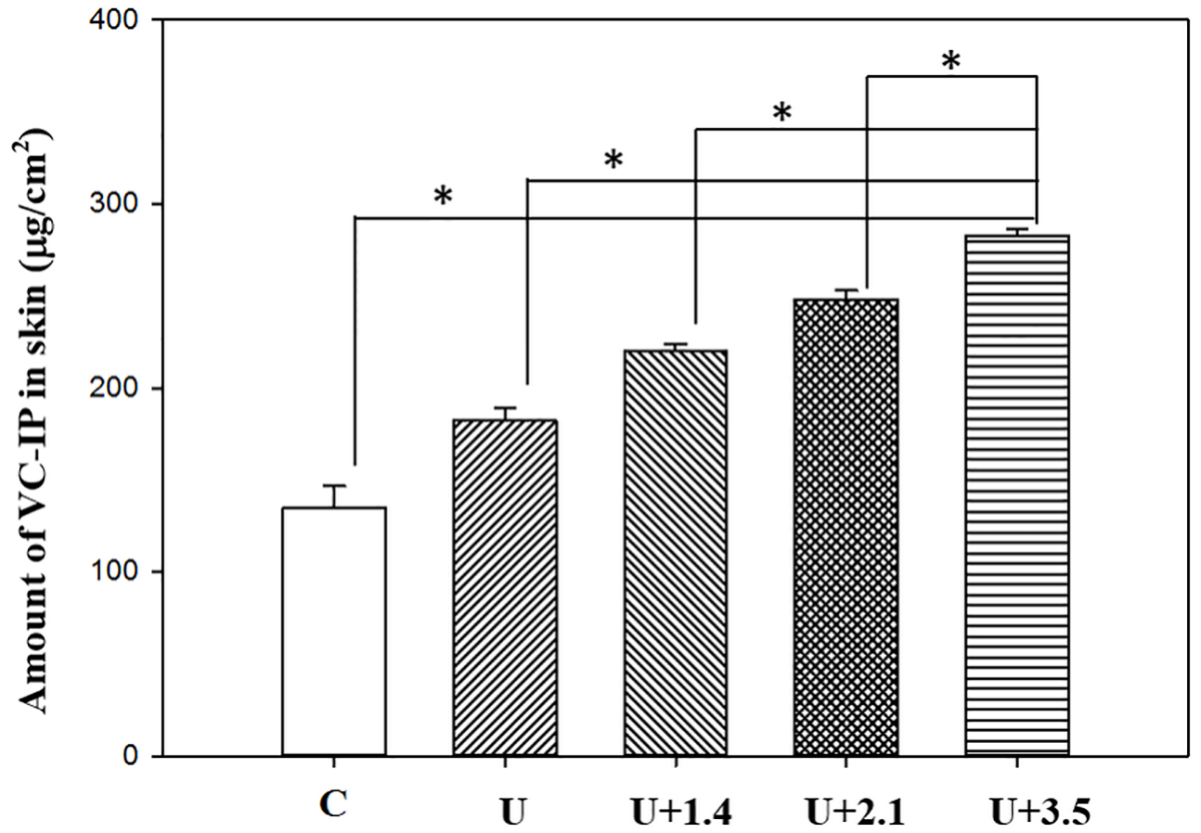
The amount of VC-IP deposited in the skin appeared to be higher in group U than in group C, and significantly so in groups U, U+1.4, U+2.1, and U+3.5. The amount of VC-IP that had permeated at 32 hours and the residual amount of the initially administered drug remaining in the skin. Data are mean and SD values.

Fig 5 shows the VC-IP concentrations in the five groups for percutaneous penetration over 32 hours as analyzed using the UV/visual spectrophotometer. The concentrations in all groups increased rapidly during the first 24 hours, reaching 135.8±5.0 μg/ml, 164.8±16.0 μg/ml, 217.9±6.1 μg/ml, 237.6±1.7 μg/ml, and 273.5±3.0 μg/ml in groups D, U, U+1.4, U+2.1, and U+3.5, respectively, and then gradually leveled off between 24 to 32 hours; at 32 hours the concentrations were 139.5±6.2 μg/ml, 179.4±5.1 μg/ml, 216.5±1.8 μg/ml, 242.6±3.4 μg/ml, and 268.3±4.3 μg/ml. The concentrations differed significantly (Bonferroni *p<*0.05) among groups D, U, U+1.4, U+2.1, and U+3.5. Combining US with MBs of different sizes resulted in 1.3–2.0 times more the penetration and deposition of VC-IP than in group D. Fig 6 shows that the amount of VC-IP deposited in the skin appeared to be higher in group U than in group D, and significantly so in groups U, U+1.4, U+2.1, and U+3.5.

### Animal treatments

**Fig 7:**
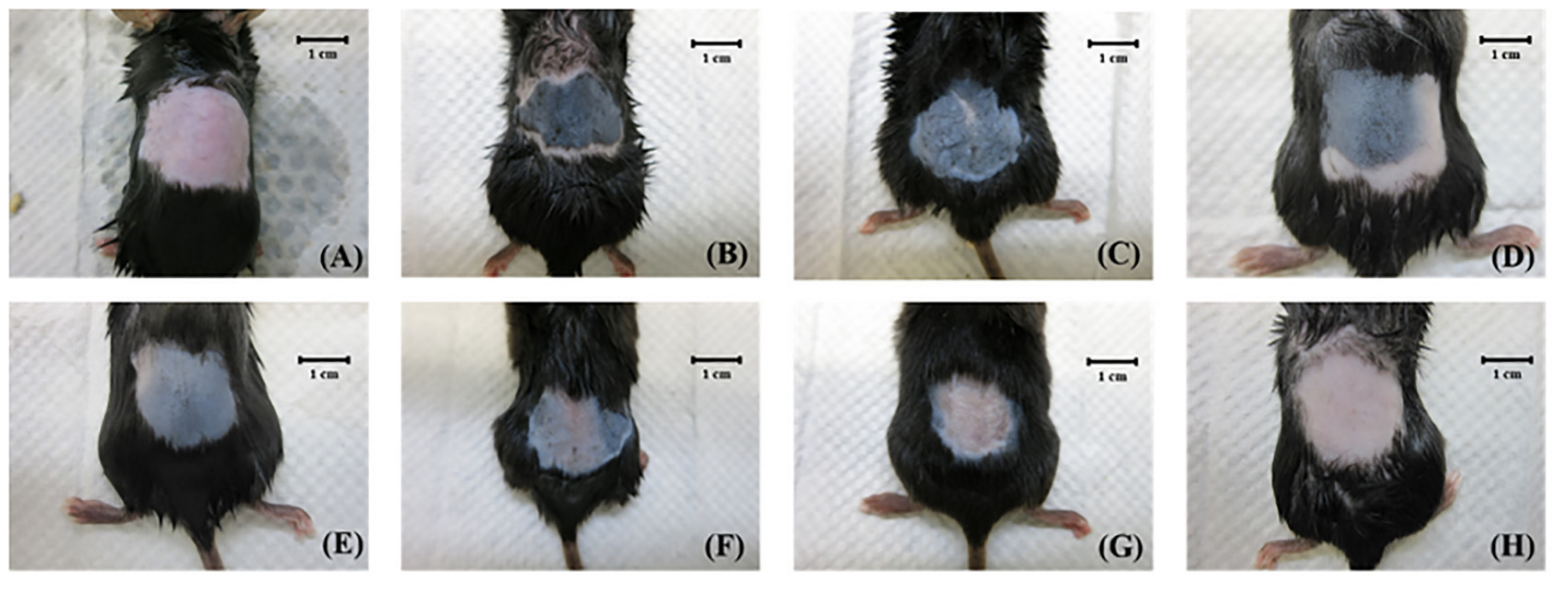
Photographs of mouse skin in a completely untreated animal (A), after UVB irradiation (B), and in groups C (C), D (D), U (E), U+1.4, (F), U+2.1 (G), and U+3.5 (H) at week 4.

**Fig 8:**
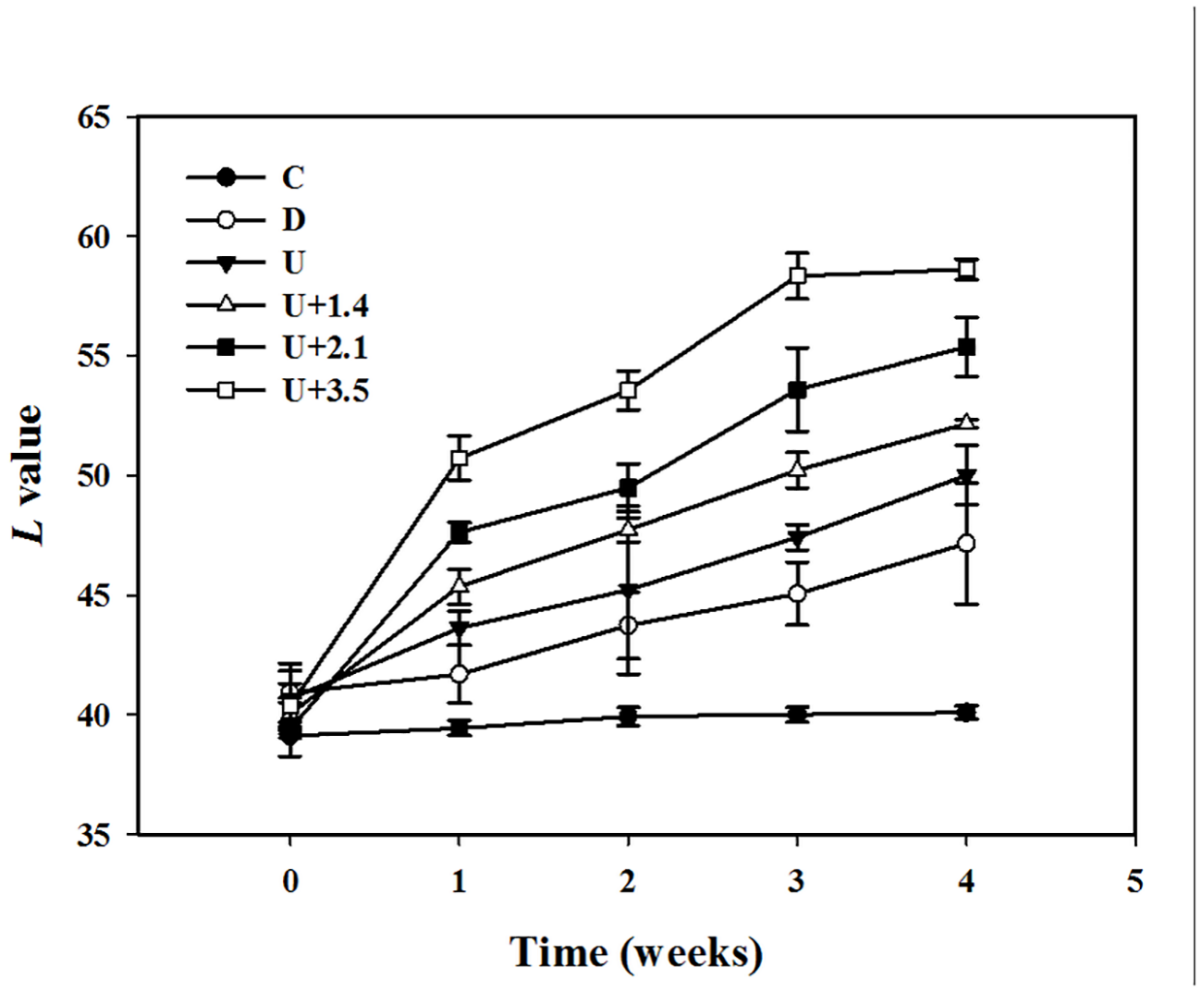
Skin-whitening effects of VC-IP on UV-induced hyperpigmentation in groups C, D, U, U+1.4, U+2.1, and U+3.5 over 4 weeks. Data are mean and SD values.

Fig 7 shows photographs of mouse skin in a completely untreated animal (Fig 7A), after UVB irradiation (Fig 7B), and in groups C (Fig 7C), D (Fig 7D), U (Fig 7E), U+1.4 μm (Fig 7F), U+2.1 μm (Fig 7G), and U+3.5 μm (Fig 7H) at week 4. The skin brightness in group U+3.5 was more effectively increased and closer to the original color compared to groups U, U+1.4, and U+2.1. Fig 8 plots the brightness (i.e., *L*) values to demonstrate the whitening effects of VC-IP on UV-induced hyperpigmentation over 4 weeks. The brightness value was around 40 in each group after UVB exposure (the possible range in the brightness value was 0–100). At week 1 the brightness value in group U+3.5 had increased by 29%. Significant differences (*p*<0.05) were obvious between each treatment group and group C, but not among groups D, U, and U+1.4 (Bonferroni *p>*0.05). At week 2 the brightness values in groups D, U, U+1.4, U+2.1, and U+3.5 had increased by 12%, 16%, 22%, 27%, and 37%, respectively. At week 3 the brightness value in group U+3.5 had increased by 50%, making it close to the original skin color, while those in groups D, U, U+1.4, and U+2.1 had increased by smaller amounts (by 15%, 21%, 28%, and 39%, respectively). At week 4 the brightness value in group U+3.5 had reached a plateau, with an increase of 51%, while those in groups D, U, U+1.4, and U+2.1 had increased by 21%, 28%, 33% and 41%, respectively. The brightness value before UVB exposure was 60.08±1.12, and after 3 weeks and 4 weeks it was only close to this value in group U+3.5.

**Fig 9:**
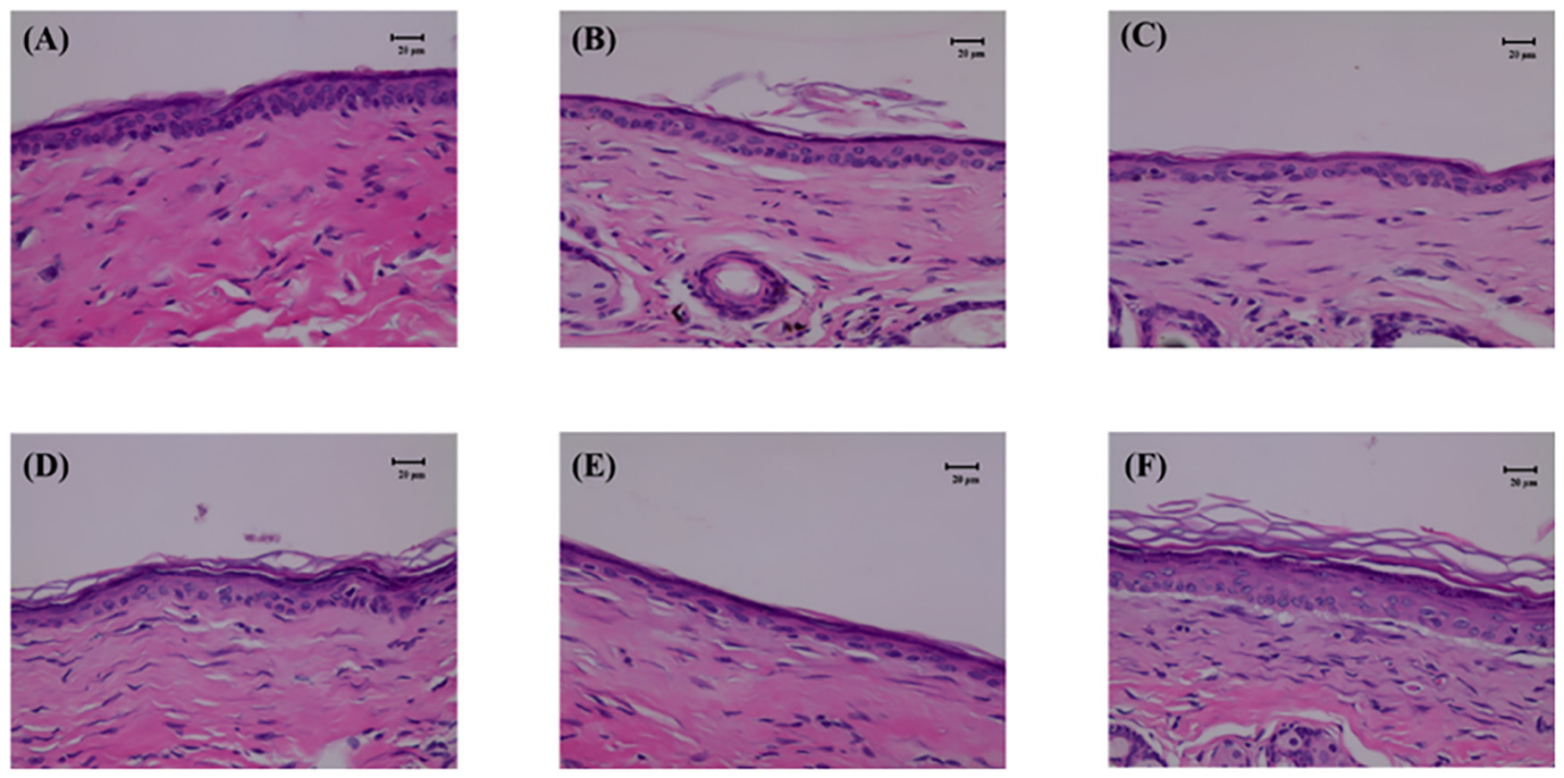
Histology images for groups C, D, U, U+1.4, U+2.1, and U+3.5 at 4 weeks. Data are mean and SD values.

The histology images in Fig 9 indicate that no skin damage was evident in any of the US treatment groups. No changes in skin structures or bilayer–bilayer interfaces were observed in any of the treatment groups.

## Discussion

This study produced MBs of different sizes (1.4–3.5 μm) by adding different concentrations of dextrose with a one-sonication-step protocol (200 W for 2 min). A previous study found that increasing the dextrose solution from 5% to 15%, and increasing the initial sonication power (250 W for 30–40 seconds) as well as the subsequent sonication power (450 W for 20–30 seconds) produced more larger-diameter MBs that were relatively uniform in size and more resistant to US lysis [22]. The present study evaluated the efficacy of applying US in the presence of MBs of different sizes to enhance TDD by inertial cavitation, since cavitation-induced collapses of MBs disrupt the lipid bilayers of the SC [23]. The penetration depths measured in the agarose phantoms indicated that the efficiency of TDD was greater for larger MBs. However, for MBs of the same size, the penetration depth was not affected by increasing the power density. In contrast, the pigskin results indicated that the penetration depth was enhanced by increasing the power density, which may be due to differences in the density composition and structure between the phantoms and pigskin. The structural changes reached a maximum at a power density of 2 W/cm^2^ in the phantoms. Since the structural and physiological mechanisms of pigskin are similar to those in human skin, 3 W/cm^2^ US was combined with 3.5-μm MBs in the experiments on *in vitro* skin penetration and *in vivo* animal treatments.

The use of the larger MBs resulted in penetration depths that were more uniform, which indicates that larger MBs can enhance the cavitation effect and thereby increase the effectiveness of drug delivery. A previous study demonstrated that VC-IP is a derivative of vitamin C, and effectively suppresses UVB-induced skin pigmentation, possibly through its antioxidative activity, and improved the limitation of stability [13]. The permeability is still limited due to the high molecular weight (1129.8 Da) of VC-IP. The SC is generally about 10 μm thick, but on the palms and soles it ranges up to 600 μm in thickness. Although the SC is an efficient barrier, some chemical substances are able to penetrate it so as to reach the underlying tissues and blood vessels. These substances are characterized by small constituent molecules (≤500 Da), lipophilicity, and effectiveness at low dosage [24]. The use of microneedle arrays has received considerable attention in recent years as a novel but minimally invasive approach for the delivery of large transdermal drugs (>500 Da) [25]. These micron-sized needles disrupt the SC and create microscale pathways that can enhance TDD. However, the choosing of basal materials for microneedles is limited, and the needles may accidentally break so that they remain in the skin [26].

In a previous study involving human skin, although significant lightening effects were observed after 1 week of application, the difference between the 3% VC-IP cream and control group became smaller after 2 weeks and 3 weeks of application [13]. This was attributed to the suntanned skin color returning to the basal level. In our experimental results for small animals, the skin brightness values averaged 40 (possible range, 0–100) in group C, and showed no significant improvement during the study period. Although the skin brightness values improved with either VC-IP or US treatment alone, these enhancements were slow, the former due to VC-IP being a fat-soluble substance and its high molecular weight. US-mediated MB cavitation increased the permeability of the lipid bilayer, especially in group U+3.5. These results indicate that US combined with 3.5-μm MBs can significantly reduce the UVB-induced elevation of melanin production, possibly through the suppression of IL-1α and PGE_2_ secretion by keratinocytes. Similar to the BBB opening, US-mediated MB cavitation was safely induced by nonlinear MB oscillations at a certain pressure threshold, and the volume of drug administered was strongly dependent on both the power density and the MB diameter [10, 11]. In their study, larger diameter bubbles and lower pressure amplitudes can determined to be safe and consistent in their associated BBB opening. It is due to the interaction between larger MBs and the US could induce the BBB opening through nonlinear oscillation, and do not cause inertial cavitation [10, 11]. Our results suggest that the skin permeability was improved significantly dependent on both the optimize power density and larger MB diameter.

## Conclusions

Combination therapy with larger MBs and US can improve the penetration depth, concentration, and efficiency of TDD of large molecules (e.g., VC-IP) relative to either the drug alone or US treatment alone. The optimal size for the MBs on the skin for delivering VC-IP is suggested to be 3.5 μm after treatment with US. During 4 weeks of *in vivo* treatment, the brightness value increased more in group U+3.5 than in the other groups after week 1, and reached a plateau phase at week 3 and week 4. The lightening effects exerted by VC-IP on UVB-induced hyperpigmentation after US combined with 3.5-μm MBs inhibit the production of melanin by active melanocytes.
